# Supplementary material for: Genomic characteristics of Salmonella enterica serovar Blockley
Source: Microbiol Spectr. 2024 Nov 14;12(12):e02048-24. doi: 10.1128/spectrum.02048-24 (PMC11619412; doi:10.1128/spectrum.02048-24)
Supplement: Supplemental figures — Fig. S1 to S3. [file spectrum.02048-24-s0001.pdf]

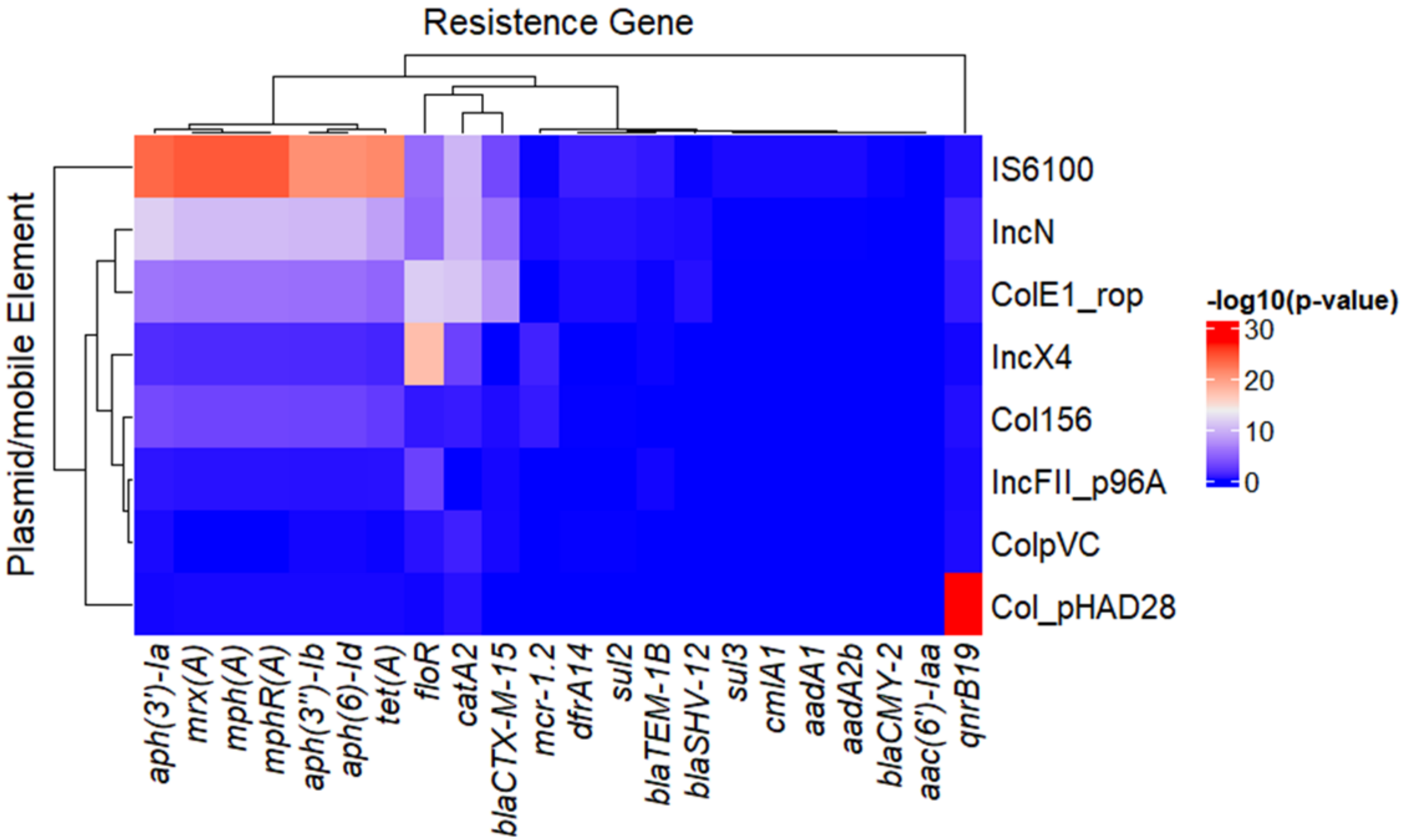

Figure S1. Heatmap based on the p-value of the Chi-square test according to the distribution of combinations between plasmid replicons/IS and resistance genes.

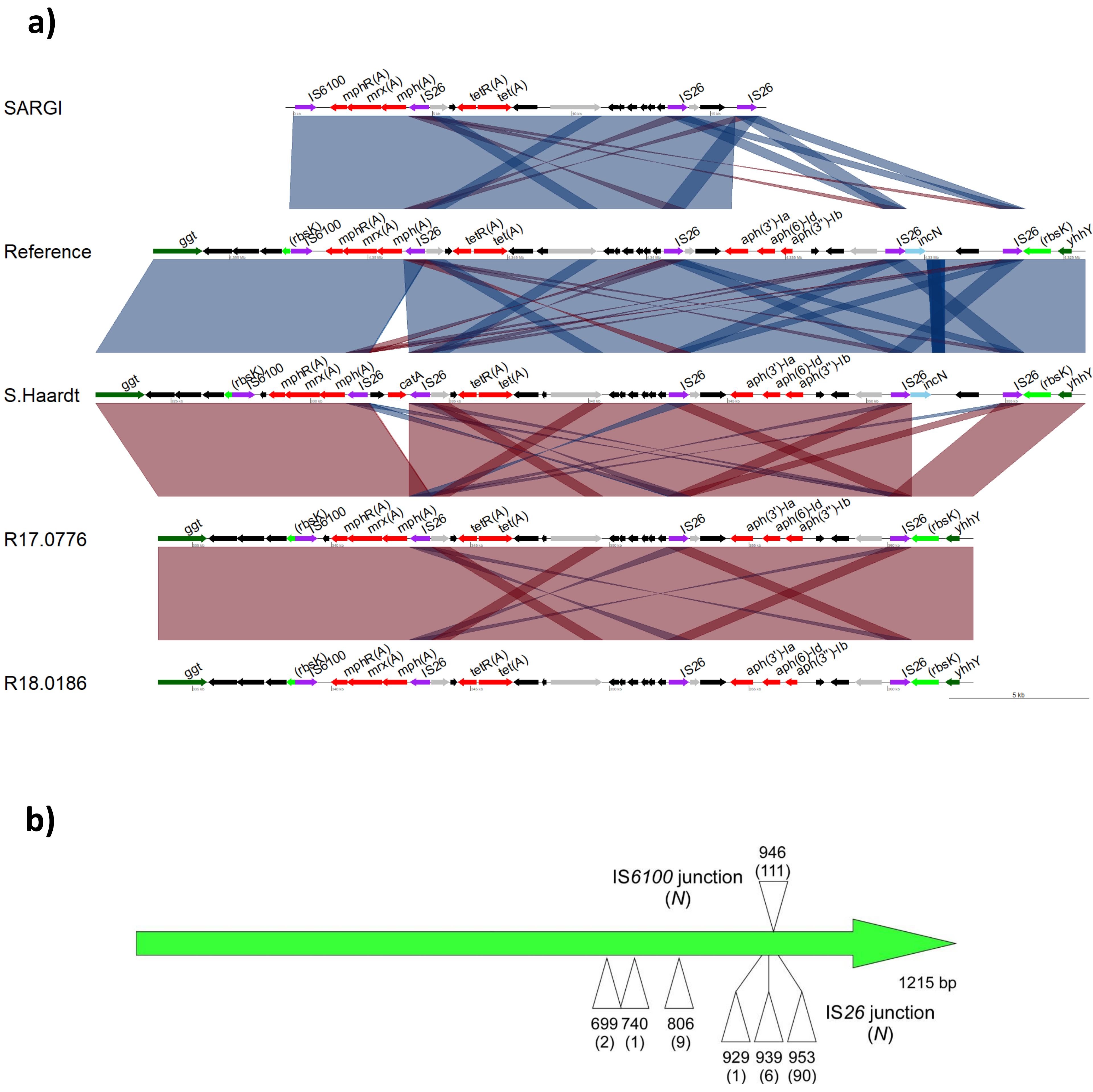

Figure S2. Genomic structure associated with SARGI. a) Alignment of SARGI-containing genomic islands. Sequences are from SARGI (GenBank accession no. KX237654), an *S.* Blockley reference strain (CP043662), an *S.* Haardt strain (AP020330), *S.* Blockley strain R17.0776 (CP100728), and an *S.* Blockley strain R18.0186 (CP100710). Arrow boxes indicate open reading frames. Resistance and related genes are colored in red, IS*26* and IS*6100* are colored in purple, conserved *ggt* and *yhhY* genes are colored in dark green, the IncN *rep* gene is colored in sky blue, and the *rbsK* pseudogene is colored in green. Gray arrow boxes indicate other tranposons. This was visualized by genoplotR. Colors of comparisons between sequences is based on default settings with a transparency; direct and reversed comparisons are colored in red and blue hues, respectively, whose gradations depends on identity between the sequences. b) Junction points of IS*6100* (upper) and IS*26* (lower) in *rbsK*.

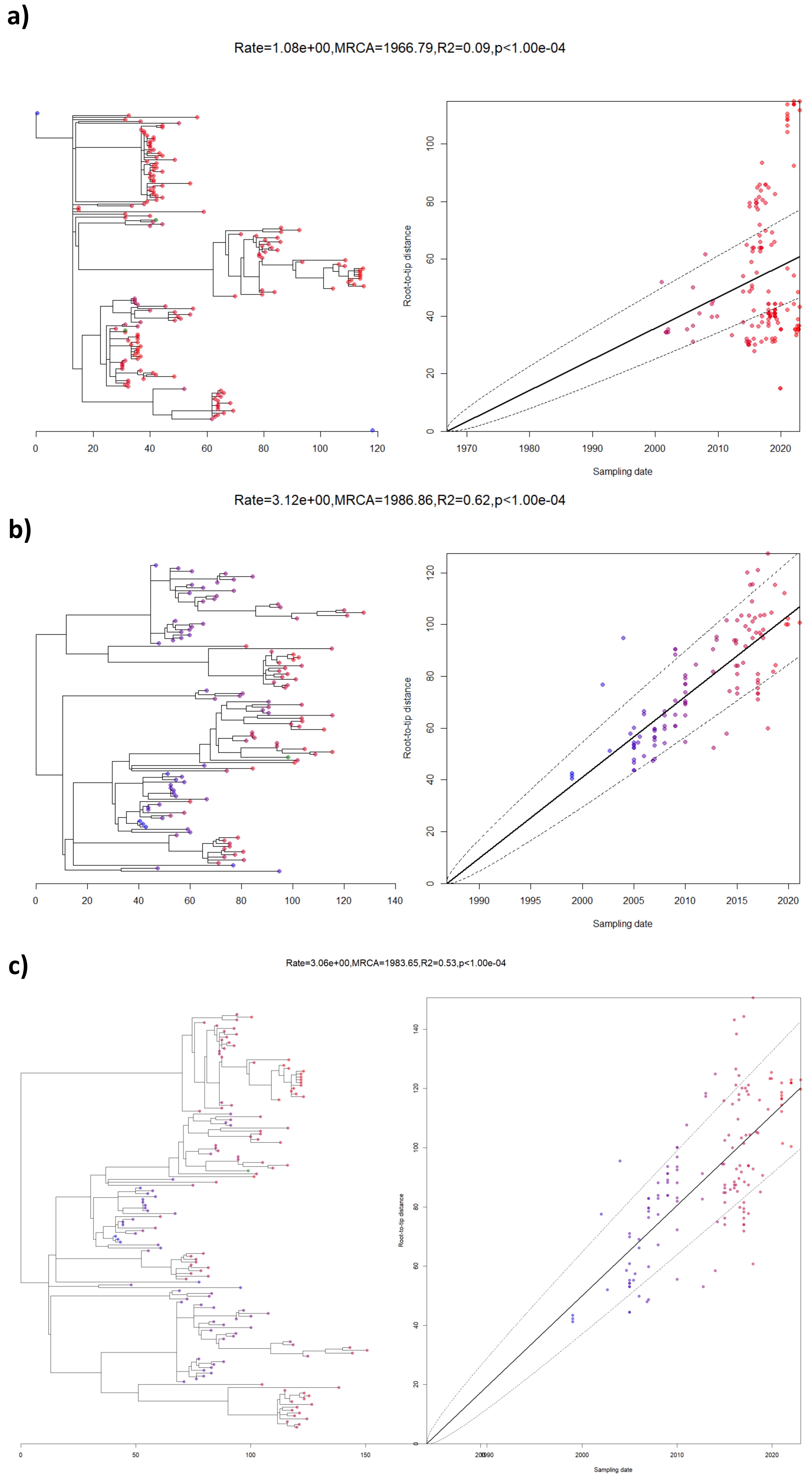

Figure S3. Initial inference of *S.* Blockley isolates by a root-to-tip analysis of BactDating. a) lineage S, b) lineage R, c) lineage R plus BAPS I.
